# Symmetric Pseudoathetosis of Hands and Feet: A Rare Manifestation of Subacute Combined Cord Degeneration With Life-Threatening Thromboembolic Risk

**DOI:** 10.1155/crnm/1862715

**Published:** 2025-08-04

**Authors:** Ramsha Siddiqui, Johanna Canenguez, Nithisha Thatikonda, Awab Elaneem, Fernandez Jorge Rodriguez

**Affiliations:** Department of Neurology, University of Texas Medical Branch and Hospitals (UTMB), Galveston 77550, Texas, USA

**Keywords:** elevated homocysteine, pseudoathetosis, pulmonary embolism, SACD, vitamin B12 deficiency

## Abstract

Vitamin B12 deficiency can cause subacute combined degeneration (SACD) by disrupting myelin synthesis, leading to spinal cord degeneration. We present a unique case of SACD featuring symmetrical pseudoathetosis characterized by involuntary, slow, and writhing movements resulting from proprioceptive sensory impairment, which disrupts the brain's ability to accurately perceive limb position and movement and pulmonary embolism due to elevated homocysteine levels. A 34-year-old male presented with chest pain, generalized weakness, and numbness in his hands and feet. Two months prior, he experienced sharp chest pain, followed by progressive numbness and weakness in his upper and lower extremities. Neurological examination revealed no nuchal stiffness, normal cranial nerve function, and impaired light touch and vibration sensation in the lower extremities. Tremulousness in the hands and feet, suggestive of pseudoathetosis, had been present for 6 months. Laboratory tests confirmed severe vitamin B12 deficiency (< 159 pg/mL), elevated homocysteine, and pancytopenia. MRI of the spine showed hyperintense signals consistent with SACD, and a chest CT revealed a large saddle pulmonary embolus. Pernicious anemia was confirmed as the cause of vitamin B12 deficiency. The patient was treated with intravenous vitamin B12, leading to significant neurological improvement. This case is the first documented instance of SACD presenting with symmetrical pseudoathetosis in all four extremities. Recognizing this rare clinical sign is essential, as it can guide early diagnosis and treatment. In addition, hyperhomocysteinemia associated with vitamin B12 deficiency is a significant risk factor for thromboembolism, underscoring the need for screening in patients with unexplained thrombotic events.

## 1. Introduction

Vitamin B12 deficiency can lead to subacute combined degeneration (SACD) by disrupting myelin synthesis, resulting in spinal cord degeneration. We present a unique case of SACD characterized by symmetrical athetosis, a rare and notable clinical feature that underscores the need to recognize this symptom in the context of SACD caused by vitamin B12 deficiency.

In addition, our case highlights the importance of screening for vitamin B12 deficiency in patients presenting with thrombosis, as elevated homocysteine levels associated with the deficiency can significantly increase the risk of thromboembolic events, which can prove life-threatening if not detected and treated in a timely manner.

## 2. Case Presentation

A 34-year-old right-handed male was transferred for evaluation of chest pain and numbness/tingling in his hands and feet. His symptoms began 2 months earlier with sharp chest pain, followed by numbness, and tingling, He subsequently developed similar symptoms in his feet, extending up to the knees. He denied urinary or fecal incontinence, saddle anesthesia, or anal sphincter changes but did report gait difficulties due to lower limb weakness. The patient is a chronic smoker, with no history of illicit drug use. He is not a vegetarian and has no history of gastric bypass surgery. The underlying cause of his vitamin B12 deficiency was identified as pernicious anemia, confirmed by the presence of anti-intrinsic factor antibodies.

Neurological examination revealed increased tone with diffuse hyperreflexia in all four extremities and clonus in the bilateral lower extremities. Muscle strength was intact with 5/5 Medical Research Council (MRC) grade in all muscle groups except shoulder elevation, which was 4/5 with some limitation with pain. On cerebellar examination, finger-to-nose testing and tremor were negative, and rapid alternating movements were intact. The patient exhibited fatigable horizontal nystagmus. Gait was initially deferred; on later assessment, the patient was able to take one to two shuffled steps but was unable to ambulate further and ultimately required a walker for mobility. The Romberg test was not performed due to significant instability, as the patient was unable to stand without assistance. Sensory examination showed impaired vibration and proprioception up to the knee level bilaterally. Slow, involuntary, writhing movements were observed in both the hands and feet, as demonstrated in Video 1.

An extensive workup was conducted to evaluate for alternative causes of spinal cord lesions. The results were negative for demyelinating disorders (anti-MOG and aquaporin-4 antibodies), infectious etiologies (including syphilis and HIV), and heavy metal toxicity. Copper levels were within normal limits at 101 μg/dL (reference range: 70–140 μg/dL). Cerebrospinal fluid (CSF) analysis showed a protein level of 41 mg/dL, cell count of 2, and negative cultures, ruling out infectious or inflammatory causes. A hypercoagulable workup was negative, including tests for antiphospholipid antibodies, Factor V Leiden, prothrombin gene mutation (Factor II), and Protein C and S activity. Laboratory tests revealed a severe vitamin B12 deficiency (< 159 pg/mL), elevated homocysteine levels, and pancytopenia. MRI of the spine demonstrated T2 and STIR hyperintense signals in the cervical and thoracic spinal cord, consistent with SACD. In addition, a CT scan of the chest identified a large saddle pulmonary embolism.

**Figure 1:**
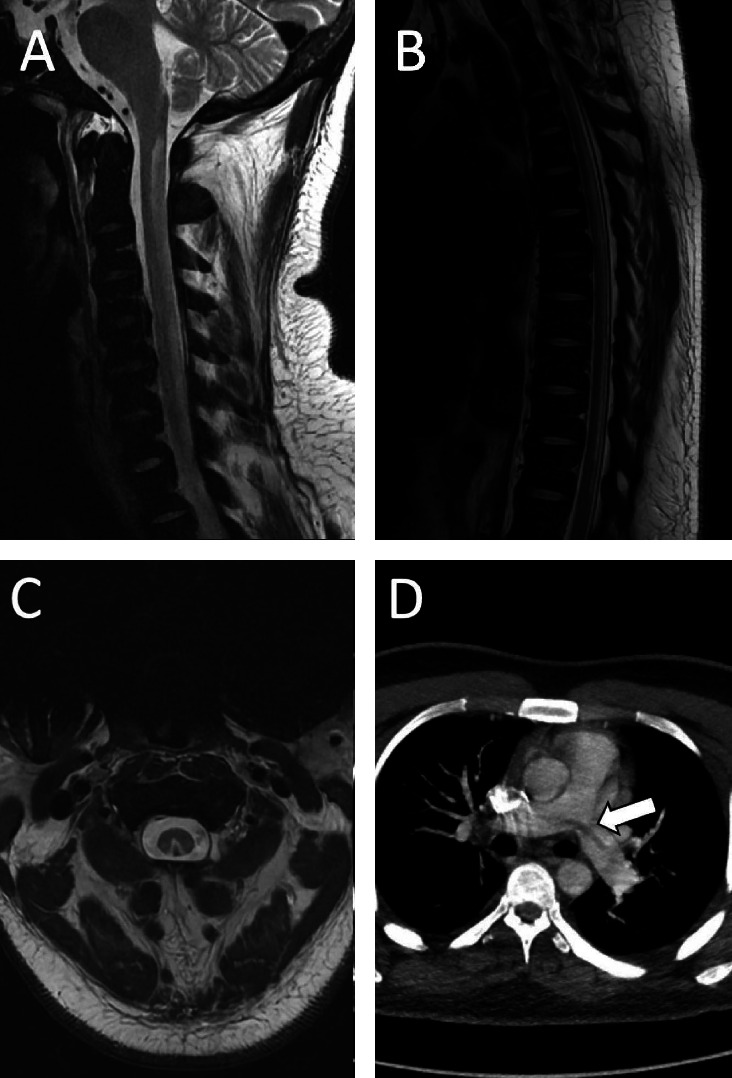
MRI spine showing T2-hyperintensities involving dorsal cervical (A) and thoracic (B) cord with inverted V-sign (C). (D) Saddle pulmonary embolism.

Relevant imaging findings are shown in Figure 1.

Intrinsic factor antibodies confirmed pernicious anemia as the cause of the patient's vitamin B12 deficiency. He was started on intramuscular cyanocobalamin (vitamin B12) at a dose of 1000 mcg once weekly, which was continued for 8 weeks until normalization of serum B12 levels. The regimen was then transitioned to monthly maintenance injections.

During his inpatient stay, the patient was also initiated on duloxetine (Cymbalta) 20 mg at bedtime for symptomatic relief and began physical therapy to address motor deficits. Additional supportive therapies included vitamin D supplementation (50,000 IU weekly), ferrous sulfate (325 mg every 48 h), and folic acid (1 mg daily).

At his 3-month follow-up, the patient reported improvement in his unsteady gait, although the involuntary movements and impaired proprioception remained largely unchanged.

The patient also initially presented with chest pain and dyspnea, though he did not require oxygen supplementation. His Pulmonary Embolism Severity Index (PESI) score was 44, placing him in the low-risk category. He was treated with a direct oral anticoagulant (DOAC) for a total duration of 6 months.

## 3. Discussion

This case underscores how a seemingly simple vitamin B12 deficiency can lead to severe complications such as pulmonary embolism and subacute combined cord degeneration. Notably, it presents with unique physical exam findings like pseudoathetosis of the hands and feet in symmetric fashion, a rare manifestation of subacute combined cord degeneration.

Degeneration of the dorsal and lateral columns of the spinal cord due to B12 deficiency is known as SACD. Typically, SACD presents with sensory ataxia, gait difficulty, and impaired vibration and proprioception in length dependent fashion. However, in our case, the patient presented with complaints of involuntary movements of all four distal extremities symmetrically consistent pseudoathetosis, which is an extremely rare phenomenon.

Pseudoathetosis is a movement disorder characterized by involuntary, slow, and writhing movements resulting from proprioceptive sensory impairment, which disrupts the brain's ability to accurately perceive limb position and movement [1]. Pseudoathetosis primarily affects the dorsal column-medial lemniscus (DCML) pathway, which is responsible for transmitting proprioceptive information which originates in the muscle fibers and joint receptors to the contralateral somatosensory cortex. Disruption at any point along this pathway can lead to proprioceptive deafferentation, resulting in pseudoathetosis.

Pseudoathetosis predominantly affects the upper extremities in symmetric fashion than the lower extremities. This is because proprioceptive fibers from the upper limbs ascend in the cuneate fasciculus of the dorsal column, while those from the lower limbs ascend in the gracile fasciculus only up to the upper thoracic cord. From there, they leave the dorsal columns to continue in the dorsolateral spinocerebellar tracts [2]. Symmetrical pseudoathetosis involving upper limbs can be seen in severe peripheral neuropathies, SACD due to B12 and nitrous oxide deficiency, and cervical spondylotic myelopathy [3]. Pseudoathetosis can also present asymmetrically, affecting only one extremity, as seen in migrated cervical discs [4] and thalamic lesions [5], which affect the limb contralateral to the injury. Although pseudoathetosis involving the lower extremities is documented, it is less common [6]. Pseudoathetosis involving all four extremities due to SACD has not been documented before.

In clinical setting, pseudoathetosis is frequently mistaken for athetosis due to their similar appearances. However, distinguishing between the two is crucial, as their underlying causes, diagnostic approaches, and treatment strategies differ significantly. Athetosis is primarily caused by damage to the basal ganglia, while pseudoathetosis is characterized by proprioceptive deafferentation. Proprioceptive testing is essential for differentiation: patients with pseudoathetosis exhibit proprioceptive loss, whereas those with athetosis do not. In addition, athetosis is often accompanied by other movement disorders, such as dystonia or chorea, depending on the extent of basal ganglia involvement. Imaging studies can also help clarify the distinction: athetosis is associated with basal ganglia lesions, while pseudoathetosis is linked to lesions in the dorsal columns, thalamus, or peripheral nerves [7]. Understanding these differences is vital for accurate diagnosis, especially when pseudoathetosis is the initial sole presentation of the underlying neurological condition.

Pseudoathetosis can be reversible in certain conditions such as cervical spondylotic myelopathy with decompressive surgery [3]. It can be persistent if the underlying neuropathy or injury is not reversible such as thalamic strokes [5] or certain peripheral neuropathies such as leprosy [8].

Another detrimental complication of B12 deficiency seen in our patient was saddle pulmonary embolism due to elevated homocysteine levels.

In United States, the prevalence of vitamin B12 deficiency is 6% under 60 years of age, which increases to nearly 20% in those over 60 years [9] with pernicious anemia accounting for approximately 75% of all vitamin B12 deficiency cases [10]. Vitamin B12 is an essential cofactor for the enzyme methionine synthase, which plays a critical role in the remethylation of homocysteine to methionine. When vitamin B12 levels are insufficient, this pathway becomes disrupted, resulting in hyperhomocysteinemia. According to a recent study, the population-attributable risk for hyperhomocysteinemia was 29.7% in individuals with vitamin B12 deficiency [11]. Hyperhomocyeteinemia is a well-known risk factor for thromboembolism, resulting in pulmonary embolism and deep venous thrombosis and according to a study, 52.1% of the patients with cerebral venous sinus thrombosis had elevated homocysteine levels attributed to vitamin B12 deficiency [12]. A study reported that 20% of the patients with vitamin B12 deficiency were found to have thromboembolic events, with hyperhomocysteinemia identified as the primary cause [13]. Similarly, another research found that 12.4% of the patients with thrombotic events exhibited elevated homocysteine levels, and in 5% of these cases, hyperhomocysteinemia was the sole identified thrombophilic factor [14].

Overall, this case highlights the importance of identifying rare and serious manifestations of B12 deficiency such as pseudoathetosis due to SACD and PE. Timely recognition correction of this deficiency is crucial to prevent life-threatening situations.

## 4. Conclusions

This case highlights two important learning points. First, while pseudoathetosis has been reported in SACD, its symmetric presentation is rare and may indicate pathology localized to the cervical dorsal spinal cord. Recognizing this uncommon physical exam finding is crucial, as it can be the sole initial symptom and guide further diagnostic evaluation. Second, hyperhomocysteinemia is associated with thromboembolism and, if left undiagnosed and untreated, can result in life-threatening complications. Therefore, screening for serum B12 and homocysteine levels should be considered in all patients diagnosed with pulmonary embolism, particularly in those without typical risk factors for venous thromboembolism.

## Data Availability

The data used to support the findings of this study are included within the article.

## Supporting Information

The video highlights the study's key finding: pseudoathetosis in both the hands and feet. Pseudoathetosis is a movement disorder characterized by involuntary, slow, writhing movements of the hands, typically resulting from a loss of proprioception. Involvement of the feet is rare. Recognizing this phenomenon is crucial, as it aids in localizing the lesion to the dorsal proprioceptive sensory pathways, particularly when pseudoathetosis is symmetrically present in both the hands and feet. Video Legend: This video demonstrates pseudoathetosis of the hands and feet (Video 1).
